# Microorganisms as Potential Accelerators of Speed Breeding: Mechanisms and Knowledge Gaps

**DOI:** 10.3390/plants14172628

**Published:** 2025-08-23

**Authors:** Sergey A. Bursakov, Gennady I. Karlov, Pavel Yu. Kroupin, Mikhail G. Divashuk

**Affiliations:** All-Russia Research Institute of Agricultural Biotechnology (ARRIAB), Timiryazevskaya Street, 42, Moscow 127550, Russia; karlov@iab.ac.ru (G.I.K.); pavelkroupin1985@gmail.com (P.Y.K.); divashuk@gmail.com (M.G.D.)

**Keywords:** speed breeding, epiphytic microorganisms, endophytic microorganisms, phytobiome, symbiosis, mycorrhiza

## Abstract

The rapid and widespread development of technology is in line with global trends of population growth and increasing demand for food. Significant breakthroughs in science have not yet fully met the needs of agriculture for increased food production and higher yields. The aim of this work is to discuss the current advancements in the application of beneficial microorganisms for crop cultivation and their integration into speed breeding technology to create optimal growing conditions and achieve the ultimate goal of developing new plant varieties. New breeding techniques, such as speed breeding—now a critical component of the breeding process—allow multiple plant generations to be produced in a much shorter time, facilitating the development of new plant varieties. By reducing the time required to obtain new generations, breeders and geneticists can optimize their efforts to obtain the required crop genotypes for both agriculture and industry. This helps to meet the demand for food, animal feed and plant raw materials for industrial use. One potential aspect of speed breeding technology is the incorporation of effective beneficial microorganisms that inhabit both the above-ground and below-ground parts of plants. These microorganisms have the potential to enhance the speed breeding method. Microorganisms can stimulate growth and development, promote overall fitness and rapid maturation, prevent disease, and impart stress resistance in speed breeding plants. Utilizing the positive effects of beneficial microorganisms offers a pathway to enhance speed breeding technology, an approach not yet explored in the literature. The controlled practical use of microorganisms under speed breeding conditions should contribute to producing programmable results. The use of beneficial microorganisms in speed breeding technology is considered an indispensable part of future precision agriculture. Drawing attention to their practical and effective utilization is an urgent task in modern research.

## 1. Introduction

Traditional breeding technologies for developing new varieties and improving crops are often slow, labor intensive, and time consuming. There is an urgent need for the speed breeding of agricultural plants that exhibit increased productivity, stability, and adaptability under varying environmental conditions, ensuring global food security amid diminishing resources. Speed breeding is an advanced plant breeding method that reduces the generation time to develop new agricultural crop varieties and accelerate breeding and research programs [1]. The acceleration of plant growth cycles and rapid generational succession is achieved by optimizing environmental conditions and managing factors such as light (spectrum, intensity, and duration), temperature, humidity, nutrient composition, and atmospheric conditions under controlled conditions [2]. By implementing speed breeding techniques in agriculture, plant breeders can effectively address food shortages caused by adverse conditions and the impacts of climate change. These speed breeding methods and technologies provide the most effective solutions for accelerating breeding processes, enhancing crop production, and promoting sustainable yield growth. The combination of speed breeding and modern breeding methods unlocks the full potential of crops [3].

To develop effective speed breeding programs, modern technologies are essential, particularly in the context of continuous population growth and the simultaneous depletion of resources. Key techniques include genomic selection, environmental optimization, CRISPR/Cas9 technology, and epigenomic tools [4], all of which significantly increase the efficiency of speed breeding. However, multifactorial and unpredictable genotype–environment interactions complicate the breeding process, as they are highly dependent on the interplay between production and breeding environments [5]. These interactions play crucial roles in determining the genetic basis for how plants adapt to new conditions [6], extending beyond abiotic factors to include biological components of the growth environment, particularly plant-associated microorganisms. Breeders should pay special attention to these multidimensional interactions, as they facilitate not only the prediction of hybrid quality but also the assessment of species versatility and adaptability [7].

**Figure 1:**
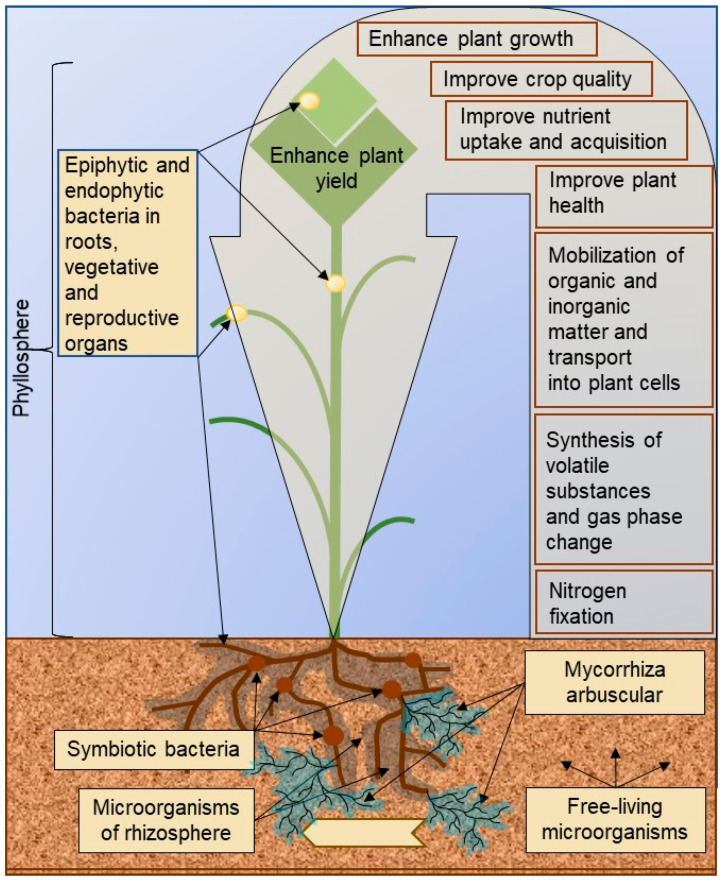
The location and impact of beneficial microorganisms of the phytobiome inside, on the surface, or adjacent to plants. The phytobiome refers to a complex ecosystem encompassing the plant host and the diverse communities of micro- and macro-organisms inhabiting its interior, surface, and immediate surroundings, all interconnected by a sophisticated network of interactions that critically influence plant health and development.

Critically, within controlled speed breeding systems, microorganisms—abundant inhabitants of both plants and growth substrates—serve as active mediators of genotype–environment outcomes. They influence plant performance through nutrient cycling (converting organic/inorganic compounds into bioavailable forms), production of growth-stimulating compounds, and modulation of stress responses. Beyond symbiotic species such as endophytes, free-living microorganisms of the rhizosphere and phyllosphere epiphytes form complex functional associations with plants (Figure 1), yet the potential of the entire microbiota remains underutilized in speed breeding regimes.

Owing to their close association with plants, these diverse microbiomes significantly influence their hosts [8]. Consequently, the application of plant-friendly microorganisms may serve as a complementary strategy to augment breeding efforts. According to the principles of natural ecosystems, the greater the diversity and number of inhabitants, the higher the order of their interaction and the more stable the ecosystem [9]. Therefore, for optimal impact, a stable microbial ecosystem must form rapidly within the breeding system. This requires sufficient microbial diversity and abundance to generate complex interaction networks. The enhancement of this artificially created environment will lead to an increase in both the number and diversity of microorganisms beneficial to plants, as well as in their biomass [9]. The potential to increase plant productivity through the ability of rhizosphere bacteria to stimulate plant growth has garnered the attention of both researchers [10,11,12] and agricultural practitioners [13,14,15,16]. However, the intricate study of beneficial interactions between plants and microorganisms under speed breeding conditions has not yet received adequate practical attention. The complex influence of plant-growth-promoting microorganisms and the multifactorial nature of their effects on various physiological processes must be considered. Factors such as plant species, bacterial strains, and growing conditions significantly influence how bacterial treatments impact these parameters [17].

Mutualistic symbioses with beneficial nodule bacteria, arbuscular mycorrhizal fungi, and diverse beneficial rhizosphere and epiphytic microorganisms (Figure 1) confer to plants the potential for accelerated growth and maturation under speed breeding regimes [18,19,20,21,22]. These plant–microbial systems foster natural interactions and create the necessary conditions for effective plant growth during speed breeding programs. Within the controlled environment of speed breeding climatic chambers, it is essential to optimize all parameters to ensure rapid and productive crop maturation. Therefore, this review emphasizes the role of microorganisms in speed breeding and highlights their practical applications. A key objective is to underscore the necessity of synchronizing plant development with their associated microbiomes to unlock additional avenues for enhancing the efficiency of this technology. Furthermore, it is crucial to raise researcher awareness of advances in beneficial microflora research, as this knowledge can enhance the rapid development of vital plant varieties under speed breeding.

## 2. Principal Aspects of Speed Breeding Technology

Plant breeding is crucial for the future of agricultural production. Most conventional breeding programs require approximately 10 to 15 years to develop varieties with enhanced traits. The most time-consuming aspect of the trait improvement process is the duration of each breeding cycle. Speed breeding is a contemporary approach that aims to rapidly increase the number of crops by optimizing environmental conditions to achieve swift generation turnover [23], ultimately addressing the global demand for food [24].

This technology involves cultivating crops under controlled environmental conditions, specifically via climatic systems. It focuses on accelerating photosynthesis and flowering, coupled with early seed harvest, by optimizing light parameters (spectrum, intensity, and photoperiod duration), temperature control, soil moisture, and the supply of specialized nutrients, along with other calibrated parameters that lead to a shorter generation time [25]. Additionally, this method may include manipulating high planting densities, employing plant growth regulators, and increasing carbon dioxide levels in growth chambers to stimulate accelerated growth from the vegetative to reproductive stages [26,27]. Minimizing the seed-to-seed cycle time reduces the life cycle of cultivated plants, promotes the rapid establishment of stable and homozygous genotypes, and facilitates the development and release of new crop varieties [2]. To shorten the period between seed formation and the beginning of the life cycle of the next generation, it is also possible to use methods such as embryo culture and overcoming seed dormancy [28]. Depending on the plant species and its photoperiod sensitivity, two to six generations per year can be achieved. Speed breeding is emerging as a valuable tool to increase the efficiency of breeding programs, reduce costs, and decrease labor intensity.

**Figure 2:**
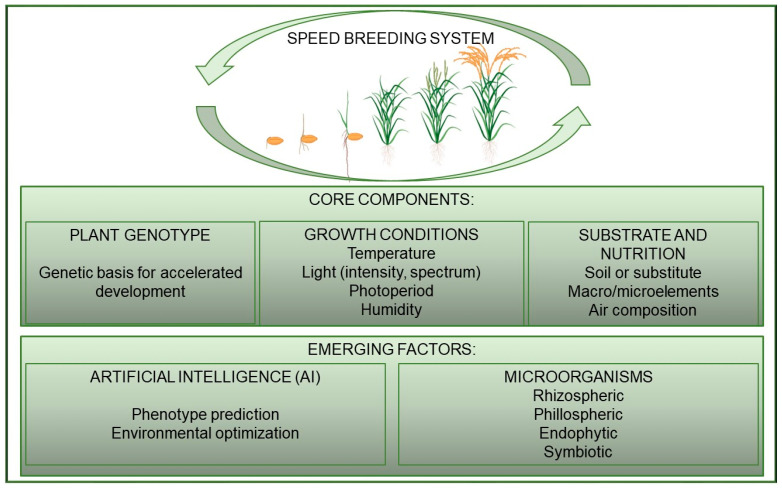
Core and advanced components of a speed breeding system. The figure outlines fundamental elements, such as plant genotype, controlled growth conditions (light intensity, spectrum, photoperiod, humidity, air composition), and substrate composition (soil or alternatives, macro/micronutrients). Emerging innovations include AI-driven phenotype prediction and environmental optimization, alongside targeted management of beneficial microbiomes (rhizospheric, phyllospheric, and endophytic microorganisms) to accelerate plant development.

Exposure to light has a fundamental effect on the development cycle of plant generations [29,30]. It unlocks their genetic potential and facilitates the development of new crop varieties with high yield potential, enhanced nutritional quality, and increased resistance to both biotic and abiotic stresses by triggering specific signaling pathways via photoreceptors. Modifying light conditions—such as intensity, spectral composition and duration (photoperiod)—directly affects photoreceptors in plants, thereby accelerating their reproductive development [31]. Photoreceptors respond to light and play a key role in regulating the natural circadian rhythm, among other physiological processes, responding to changes in environmental conditions [32]. Speed breeding enables rapid generational changes in neutral and short-day plants due to extended illumination, as well as in long-day plants by initially using a shortened photoperiod to promote vegetative growth before controllably inducing flowering [31,33]. The ability to control growing conditions year-round allows for the simulation of various agroecological conditions [34] and the efficient application of phenotyping and molecular genomics tools to accurately introduce new traits and increase the overall rate of crop improvement [35]. Furthermore, speed breeding protocols can be tailored to specific plant life cycles, including winter/spring types, annuals, biennials, and perennials [31]. The various components of speed breeding technology are summarized in Figure 2.

This methodology can also integrate additional technologies to further improve efficiency, including various techniques that expedite plant breeding [4]. The integration of genotypic, environmental, and phenotypic data [36] enables the rapid identification of parent plants with superior agronomic traits [37]. This is achieved through marker-assisted selection [38], to produce crops with increased yields and other desirable characteristics during subsequent hybridization. The monitoring and analysis of phenotypic data can facilitate the identification of quantitative trait loci (QTLs) associated with desired traits [39,40]. Therefore, combining speed breeding with modern tools, such as genome editing and high-throughput genotyping and phenotyping systems, significantly accelerates the development of new plant varieties.

Speed breeding conditions differ significantly from those of natural environments and can induce various stresses, including heat, oxidative, and nutritional stresses, which must be understood and managed effectively. The introduction of microorganisms into speed breeding systems can induce short-term stress in plants due to the activation of immune responses and resource competition. Furthermore, specific speed breeding stressors (heat, reactive oxygen species (ROS), nutrient imbalance) alter root exudate profiles. This can disrupt microbial colonization kinetics and symbiotic signaling pathways, potentially reducing symbiosis efficacy [41]. However, these risks are manageable through the use of adapted microbial consortia, rhizosphere engineering optimization, and dynamic microenvironmental control. Properly selected microbes not only minimize additional stress but also enhance plant resilience to speed breeding conditions. Consequently, strain selection must prioritize compatibility with speed breeding stressors, low potential to trigger strong immune responses, and adaptability to dynamic rhizosphere chemistry [42,43].

Accelerated growth may lead to the rapid local depletion of nutrient reserves. Therefore, optimized nutrient delivery systems are essential for successful speed breeding. Given the well-documented role of beneficial rhizosphere microorganisms in enhancing nutrient acquisition under conventional conditions [44,45], their potential to improve nutrient use efficiency in speed breeding systems warrants further investigation. Prolonged exposure to light can increase ROS production in speed breeding systems. Elevated ambient or leaf surface temperatures, potentially exacerbated by specific light spectra, can further contribute to oxidative stress. Antioxidant systems are vital for plant resistance to oxidative stress. Crop microbiomes reinforce these defenses through multiple mechanisms, including the direct production of metabolites and antioxidant enzymes, priming of host antioxidant pathways (e.g., via upregulation of APX, GPX genes), and reduction in ROS generation by improving photosynthetic efficiency [46,47,48]. Furthermore, the closed environment of climatic chambers creates conditions conducive to the accumulation and proliferation of pathogenic microbiota, posing a significant risk of contamination. This necessitates rigorous disinfection protocols for all inputs (water, growth substrate, air, seeds) as well as the chamber interior and equipment (shelves, lamps, etc.), typically employing methods like ozonation and UV treatment.

Intensive artificial lighting and long photoperiods in speed breeding [4] can induce oxidative stress, triggering the accumulation of ROS [49] and plant overheating [50]. These abiotic stressors, along with pathogenic microbiota, can reduce the efficiency of speed breeding by damaging plants’ reproductive structures—male (pollen) and female (embryo sac) gametes—as well as the nourishing tissues (tapetum). This leads to decreased pollen and ovary fertility, impaired gametophyte development, and, consequently, a significant reduction in seed set [51]. Under these conditions, plant-growth-promoting rhizobacteria (PGPR) and endophytic fungi activate antioxidant defense mechanisms by upregulating the expression of antioxidant genes (SOD, CAT, APX, and others) and producing antioxidant compounds [49,52]. By mitigating oxidative stress, the reproductive performance of plants in speed breeding can be improved. Thus, the use of microorganisms to enhance plant fitness may shorten generation turnover [4].

In relatively confined spaces such as growth chambers, persistent pathogenic microbiota can accumulate and rapidly proliferate under favorable conditions [2,31]. High humidity, temperature, planting density, and frequent watering in closed speed breeding systems create ideal conditions for pathogens (particularly fungi such as *Fusarium* spp., *Botrytis cinerea*, and other disease-causing agents). These pathogens attack reproductive structures (flowers, ovaries), causing sterility, abortion, or the production of nonviable/toxic seeds, negating the benefits of speed breeding cycles. For example, in wheat, infection by *Fusarium graminearum* (causing *Fusarium* head blight) under controlled conditions can lead to spike sterility and yield losses of 40–50%. Seed treatment with *Bacillus velezensis* (a producer of lipases and chitinases) suppressed fungal growth in such systems, reducing spike infection by 70% and enabling yield recovery to 85% of healthy control levels. Head blight caused by *Fusarium graminearum* led to spike sterility and a 40–50% yield loss. Seed treatment with *Bacillus velezensis*, which produces lipases and chitinases, suppressed fungal growth, reducing spike infection by 70%. Yield subsequently recovered to 85% of the healthy control level [53]. Similarly, *Pseudomonas chlororaphis* demonstrates potent antifungal activity against *Botrytis cinerea*, acting as a biocontrol agent that inhibits infection and restores normal seed set [54]. The presence of beneficial microorganisms secreting antibiotics and other compounds antagonistic to phytopathogens (lipopeptides, polyketides, and antifungal metabolites) [55] can mitigate the negative impact of pathogens [56,57,58] in speed breeding systems. Moreover, in the long term, pathogen-antagonistic bacteria hold a significant advantage, as they also evolve their own mechanisms to counteract pathogens. Currently, there is a wide range of commercially available biocontrol agents for the control of plant pathogens [59,60].

Artificial intelligence (AI) deserves particular attention as a comprehensive, fundamental, and transformative method that has already proven effective in speed breeding technology. AI’s ability to rapidly process, integrate, and extract meaning from vast, complex datasets is indispensable for realizing the full potential of speed breeding. AI has long been established as a key tool in accelerating plant breeding [61,62,63]. The generation of vast and complex datasets from plant “omics” studies [64], the acquisition of whole-genome sequences of target species using next-generation sequencing (NGS) [33], and the integration of marker-assisted selection (MAS), genome-wide association studies (GWAS), and transcriptomics and proteomics data demand innovative solutions. AI plays a pivotal role in processing these massive datasets and making optimal breeding decisions under speed breeding conditions [36].

The application of AI in speed breeding relies on data analysis using machine learning and deep learning algorithms based on neural networks [40], which are capable of handling complex nonlinear relationships [40]. This is particularly crucial for predicting disease resistance, productivity, and stress adaptation traits.

AI systems leveraging models like the Soil–Plant–Atmosphere Continuum (SPAC) are capable of detecting subtle plant responses to soil and atmospheric conditions [33,64]. Modern breeding programs aim to develop “next-generation AI” that can analyze the breeding value of genotypes while accounting for dynamic environmental changes [65].

Artificial intelligence has proven highly effective in speed breeding across multiple crop species, as demonstrated by recent studies. In soybean (*Glycine max*), AI-enabled phenotyping significantly improved stress tolerance analysis [64]. For wheat, deep learning models achieved a remarkable prediction accuracy of 89% for yield and 82% for drought resistance during early growth stages under speed breeding conditions with extended photoperiod and enhanced light intensity [66]. The integration of AI genetic algorithms with speed breeding and CRISPR-based genome editing in rice has reduced breeding cycles from six to eight generations to just three to four while maintaining selection accuracy [67]. Particularly innovative is the application of NSGA-III genetic algorithms to optimize light spectra (red/blue ratios) and temperature regimes in maize, which cut generation time from 90 to 56 days while improving energy efficiency by 35% [68]. Furthermore, AI-driven transcriptomic analysis of rice identified 17 candidate salt-tolerance genes, reducing the QTL detection time from six months to merely two weeks [69]. These breakthroughs demonstrate AI’s transformative potential in accelerating plant breeding while maintaining or improving precision.

Thus, artificial intelligence plays a pivotal role in speed breeding technology by accelerating the development of new cultivars through the automated processing of large-scale genomic, phenotypic, and environmental datasets; the prediction of valuable traits (stress resistance, yield potential) based on complex correlations; and the optimization of genotype selection using high-precision, high-throughput phenotyping. The ongoing refinement of AI methodologies promises to unlock new possibilities for creating climate-resilient and high-yielding crop varieties. This represents a critical advancement given the challenges of climate change and growing global food demand.

## 3. Rhizosphere Microbiome: Mechanisms of Plant Growth Promotion and Stress Mitigation in Speed Breeding

The interactions between plants and microorganisms result in the exchange of numerous compounds that significantly influence one another, ultimately shaping the rhizosphere microbiome. Plants have evolved the ability to modulate the composition and activity of their microbiota through the secretion of signaling molecules and other compounds [44]. Exudates released by plant roots serve as selective substrates that foster specific microbial associations. Moreover, closely related plant species host similar microbial populations [8,44]. Plant exudates play a critical role in determining the composition and function of microbial communities in the rhizosphere, influencing plant health and nutrient availability. The connection between plants and rhizospheric soil microbes is functionally dynamic, and environmental changes are perceived even at subtle levels, triggering a cascade of corresponding stress responses that enhance plant resilience to perceived challenges [52]. Under different stresses, plant signals vary, altering the types of rhizobacteria colonizing the roots [70]. Root exudates are genetically regulated, thereby predetermining high variability in exudate profiles across plant species, genotypes, developmental stages, environmental conditions, and biotic contexts. This, in turn, leads to genotype-specific rhizobacterial communities for different plant genotypes [71]. Among the diverse array of microorganisms, many establish beneficial associations that promote plant health and stimulate growth [8]. These plant-growth-promoting rhizobacteria, genera such as *Agrobacterium*, *Azospirillum*, *Bacillus*, *Erwinia*, *Flavobacterium*, *Paenibacillus*, *Pseudomonas*, and *Streptomyces*, do not form symbiotic structures. Furthermore, their interaction with plants is not species- or strain-specific.

Beneficial rhizosphere microorganisms serve as critical enablers of speed breeding by reducing generation time and mitigating system-specific stresses through targeted physiological interventions. They are capable of synthesizing a wide variety of bioactive compounds, including hormones, enzymes, and vitamins, that stimulate plant growth, thereby maximizing the plant’s potential and improving efficiency under speed breeding conditions [9]. Their primary value lies in accelerating plant development via the production of growth regulators and phytohormonal manipulation [44]. Plant-growth-promoting rhizobacteria (*Pseudomonas, Bacillus, Azospirillum*) produce auxins (particularly IAA), cytokinins, and gibberellins that directly stimulate cell division, elongation, and developmental transitions essential for rapid cycling [72,73,74]. Concurrently, bacterial ACC-deaminase activity (notably in *Rhizobium* and *Pseudomonas*) reduces ethylene-mediated growth inhibition under stress, promoting root expansion critical for accelerated resource capture [75]. These hormonal shifts enable compressed phenological phases—a cornerstone of speed breeding efficiency [76].

In addition to releasing hormones which directly influence plant growth [77,78], microorganisms also exert indirect effects by stimulating hormone production within the plant and degrading hormones that inhibit plant growth [79,80,81]. These changes can be elicited by volatiles synthesized by microorganisms [82]. Consequently, alterations in plant hormonal status can result from either the consumption or production of hormones by microorganisms or from alterations in hormone metabolism within the plants themselves [76]. Such hormonal changes can accelerate growth and development, essential for expediting development when implementing speed breeding.

Nutrient acquisition is critically enhanced to support high metabolic demands under rapid growth. PGPR solubilize immobilized phosphorus [83,84], fix atmospheric nitrogen [81], and increase iron/zinc bioavailability through siderophore production [85,86]. Crucially, microbially induced root architectural changes—including increased branching, length, and hair density via phytohormone signaling—significantly amplify nutrient and water uptake capacity [84,87]. This prevents local resource depletion during intensive cultivation cycles [9]. By altering both the structural and functional characteristics of the plant, microorganisms contribute to healthy plant physiology [88], promoting the dissolution and enhanced absorption of poorly available nutrients.

Microorganisms provide indispensable stress buffering against speed breeding constraints. Under prolonged high-light exposure, they mitigate oxidative damage by enhancing host antioxidant systems (e.g., priming APX/GPX expression) and directly scavenging ROS [46,47,48]. For heat stress inherent in controlled environments, specific PGPR induce heat shock proteins, stabilize root biofilms, improve moisture retention via exopolysaccharides (EPS) [89], and modulate stress hormone balance (e.g., ABA reduction) [90,91,92,93,94,95]. Equally vital is their biocontrol function [75]; antibiotic synthesis, the production of lytic enzymes (chitinases, glucanases), and niche competition suppress pathogens prone to proliferation in enclosed systems [72,96,97,98]. Microorganisms positively affect plants by strengthening the immune system of cultivated species [99], protecting against and inhibiting the reproduction of plant pathogens [72], and suppressing disease [96]. The rhizosphere and phyllosphere microbiota act as immune triggers, elevating concentrations of defense compounds and secondary metabolites. Plants maintaining balanced carbon allocation sustain active immune readiness, enhancing resistance to pests.

Water relations optimization further supports rapid growth kinetics. Microbial auxins promote deep root architectures [87], while EPS secretions enhance soil moisture binding and protect root integrity [100]—both critical under accelerated transpiration rates [17].

Free-living beneficial rhizosphere microorganisms possess the potential to accelerate plant growth and development, enhance nutrient use efficiency, and improve resistance to biotic and abiotic stresses and diseases. However, co-optimized plant–microbe partnerships are needed to ensure sustainable improvements in agricultural productivity and food quality [71,101,102]. Critically, under field conditions, unlike in speed breeding growth chambers, different external factors are present, which may alter the effects of specific beneficial microorganisms [71,103].

For practical implementation, tailored microbial consortia integrating complementary functions (nutrient solubilization, stress protection, growth promotion) outperform single strains in resilience and efficacy [104,105,106]. Future advances require a deeper understanding of how these microbial functions compress vegetative/reproductive phases under speed breeding regimes (altered photoperiods, thermal profiles, rapid generational turnover). Deciphering the genetic basis of plant–microbe interactions will enable precision microbial tools for next-generation speed breeding pipeline systems [4,107,108].

## 4. Symbiotic Systems in Speed Breeding Technology

The soil microbiota and symbiotic relationships are crucial for speed breeding, enhancing nutrient uptake, growth regulation, and environmental resistance to promote plant health and the rapid production of stable yields [4,109,110,111]. Owing to their unique capacity for symbiotic nitrogen fixation, legumes (*Fabaceae*) are vital for sustainable crop production [112]. Speed breeding enables up to five generations annually in grain legumes, significantly accelerating breeding cycles compared to traditional methods [113]. This intensification critically relies on two mutualisms; legume–rhizobial symbiosis with bacteria (various genera including *Bradyrhizobium*, *Mesorhizobium*, and *Rhizobium* [114,115]), which provides bioavailable nitrogen under intensive growth conditions, where host-specific rhizobial strains are mandatory for effective nodulation under accelerated growth conditions [2,70], as incompatible pairs reduce yields by 25–40% [116], and arbuscular mycorrhizal fungi (fungi *Glomeromycota* [117]), which enhance the availability of phosphorus and other nutrients. These associations increase nitrogen availability and phosphorus solubilization/uptake [77,118], underpinning plant health in accelerated growth regimes. Under speed breeding conditions, successful nodulation requires specific rhizobial strains compatible with host plants; compatible high-efficiency strains are essential for optimal yield, as their absence drastically reduces productivity [116]. Consequently, substrate inoculation with productive strains is imperative [119]. Breeding legumes for enhanced symbiosis efficiency [117] and nodulation and nitrogen fixation [120]—using markers for nodulation/nitrogen fixation [121,122]—is key to maintaining developmental synchrony in speed breeding. Notably, yield-focused selection indirectly improves nitrogen fixation [123], though polygenic constraints remain challenging [121]. Thus, tailored microbial partnerships are operationally necessary for phase transitions and high-yield stability in speed breeding [113,116].

Since nodulation and nitrogen fixation in legumes are quantitatively heritable traits [124], this information is important in the selection of parents in the breeding process. Therefore, legume breeding programs should pay more attention to plant–bacterial symbiosis to effectively translate legume growth information at the gene level into a significant increase in productivity [120]. For speed breeding conditions, the rate of establishing productive symbiosis also becomes a fundamental quality. Optimizing legume–rhizobial symbiosis (e.g., via strain selection or host genetics) to enhance nitrogen fixation accelerates seed maturation in speed breeding, ensuring rapid generational turnover. To improve legume production, future efforts should focus on developing genotypes with high compatibility with rhizobial strains [116]. Consequently, breeding programs that utilize DNA marker technology to assess the stability and efficiency of symbioses and employ genomic selection in speed breeding are needed. In this context, elucidating the genomic regions and molecular mechanisms controlling nodule formation and efficient function is essential for optimizing yield stability and quality, thereby enhancing speed breeding efficacy.

Since the rate of N_2_ fixation satisfies almost all plant nitrogen requirements, the benefit of a program aimed at improving this process in grain legumes can be significant [120,125]. For example, when pea genotypes effectively interact with beneficial soil microorganisms, complex inoculation has an effect comparable to that of the application of a full dose of mineral fertilizers [126,127,128]. The results of research on the development of breeding materials under the rapid generation advancement protocol for chickpea crops [129] emphasize the critical role of nodules in maximizing the yield of this crop and the significant yield losses associated with the absence of nodules. However, no direct evidence comparing the cultivation of plants with and without inoculation with symbiotic bacteria under speed breeding conditions can be found in the literature.

Works on legume cultivation under speed breeding technology usually include inoculation with symbiotic bacteria, despite the use of nitrate nitrogen in the experiments. For instance, soybean experiments use Legume Technology Ltd.’s (Nottinghamshire, UK) “Soya Bean Inoculum” to allow rhizobia nodule formation. This active symbiosis of nitrogen-fixing microorganisms is essential for speed breeding conditions [113]. Similar conditions were provided in pea [31,113] and chickpea studies, where seedlings were inoculated with *Rhizobium* spp. [31]. Thus, under speed breeding conditions, nodule bacteria are an obligatory component of experiments for different species of grain legumes.

In host–rhizobia interactions, some strains perform better with certain varieties of legumes than others, and such differences related to N_2_ fixation have been demonstrated for almost all legumes [130]. An understanding of the basic mechanism of rhizobial survival in soil and factors affecting symbiosis is crucial. The careful selection of inoculant strains is necessary for legume growing conditions, especially in speed breeding [9]. Improving individual parameters, such as symbiosis efficiency, under speed breeding conditions faces potential trade-offs. Competition for energy and nutrients means that enhancing one trait might compromise others, particularly given the paramount importance of minimizing the seed-to-seed generation time.

## 5. Arbuscular Mycorrhizal Fungi (AMF)

**Figure 3:**
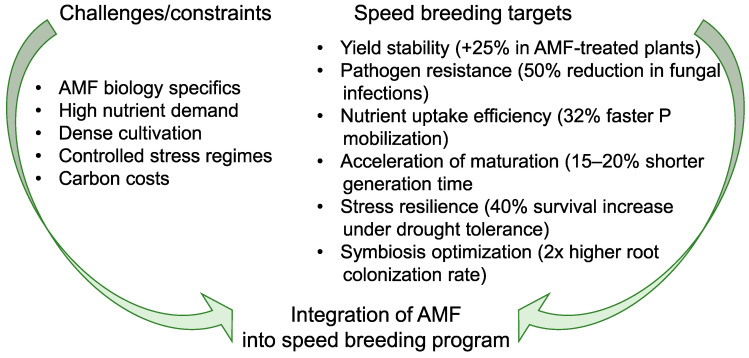
Challenges and targets for integrating arbuscular mycorrhizal fungi (AMF) into speed breeding systems to accelerate crop breeding cycles. The figure outlines key biological constraints of AMF and their implications for speed breeding. Successful integration of AMF could bridge these challenges to achieve faster and more sustainable crop improvement [152,159,160,161,162,163,164].

Arbuscular mycorrhizal fungi (AMF) form ancient, ubiquitous symbioses with >80% of vascular plants [131], inducing root cellular changes analogous to nodulation [132,133,134,135,136,137]. In speed breeding systems, AMF critically enhance sustainability under accelerated growth regimes by optimizing nutrient acquisition through hyphal networks that supply hosts with immobile nutrients like phosphorus via polyphosphate transport [138,139], offsetting high nutrient demands during rapid generational turnover [18,72,140,141,142,143,144] (Figure 3). They concurrently enhance stress resilience by improving water status, mineral nutrition (N, Mg, Ca, K, Cu, Zn, and Fe [18,145,146]), and oxidative/osmotic balance [147], mitigating abiotic stresses intensified by controlled-environment speed breeding [72,118]. AMF further reduce pathogen susceptibility by conferring resistance to root nematodes [148,149], *Verticillium* wilt [150], bacterial pathogens [151], and root rot (e.g., *Aphanomyces euteiches* in legumes via *Rhizophagus irregularis* [152]), minimizing crop losses in dense, rapid-cycle cultivation [153]. While AMF consume ≤ 20% of host photoassimilates as hexoses metabolized to storage compounds [154,155], this carbon cost represents a worthwhile investment given yield stability: AMF boost photosynthesis and secondary metabolite production [156], directly supporting high-throughput seed maturation in speed breeding. Host-specific AMF efficiency variations across crop species and genotypes [157,158] underscore the need for tailored symbiont selection in breeding programs to maximize speed breeding outcomes.

## 6. Endophytes

Endosymbionts—including bacteria, fungi, algae, viruses, and endophytes—inhabit plant tissues without negatively impacting their function or development. They colonize internal tissues, penetrate seeds and roots, and persist through much of the host’s life cycle. Endophytes are of interest in speed breeding technology due to their ability to enhance growth and nutrient uptake, alongside their positive effects on plant health under biotic and abiotic stresses [165,166].

Microorganisms living in plant tissues can significantly impact plant growth rates, including with regard to stress tolerance [167], providing benefits such as increasing mineral availability and producing antioxidants [168] and phytohormones [169] that trigger responses to abiotic and biotic stimuli [170]. Thus, endophytes that affect plant health in a positive way may play a crucial role in shortening the generation time in speed breeding.

Endophytes tend to be completely dependent on micro- and macronutrients supplied by host plants, as has been shown for *Neotyphodium*/*Epichloë* spp. [137]. However, very little is known about the mechanisms of metabolite exchange or specific transporters of sugars or amino acids [118]. A comparison of infected and uninfected plants revealed significant decreases in nitrogenous compounds (nitrate, asparagine, proline, proteins, certain fiber components) and parallel increases in soluble carbohydrates and organic acids (malate, quinate, shikimate, and phenylpropanoids) [118]. This metabolic shift, including of stress-related metabolites, may be characteristic of symbiotic associations and include pathogenic interactions [137]. The signaling processes between endophytes and their hosts are less understood in contrast to those between rhizobia and arbuscular mycorrhizal fungi. However, roles for reactive oxygen species and potentially iron in the regulating of fungal growth within plants have been demonstrated [118,171]. A shift from mutualism to pathogenicity, associated with fungal proliferation, early plant senescence, and the downregulation of fungal secondary metabolites, may occur [118]. This could potentially negatively impact cycle completion under speed breeding conditions.

The possibility of endophyte transfer through seeds to the next generation was shown earlier by Dunleavy [172]. Knowledge of the interactions between seeds and their endophytes remains limited. Seed-transmitted native endophytes are hypothesized to affect seed viability and have great potential to significantly influence germination efficiency and seedling growth [173,174,175,176,177,178,179]. Seeds therefore serve as a niche for microorganisms during germination and early growth to colonize the newly developing plant [180], and seed-mediated transmission can ensure their persistence in subsequent generations [181,182]. Pathways for such endophyte transmission from parent plants to seeds include a pathway via xylem translocation in which the flower stigma is used as an entry point [174,182,183,184,185,186].

Endophytes may be functionally important for seed germination under suboptimal conditions, as demonstrated by studying the germination viability of soybean seeds [180]. The diversity of cultivated endophytic bacteria [180] can be affected by various environmental conditions and agricultural production methods [187], including the use of various fertilizers [188]. Plant domestication can lead to the loss of beneficial endophytic microorganisms, potentially replacing them with less compatible or pathogenic ones. This shift within the endobiome can interfere with plant growth [167]. Thus, modulating the seed microbiota to improve germination and stimulate yield growth may be very important under speed breeding conditions.

## 7. Epiphytes and Endophytes of Phyllosphere

The aboveground parts of plants are also inhabited by diverse microbial communities [189], including epiphytes existing on the surface and endophytes which penetrate. The role of the root microbiota in plant life is much better understood than that of aboveground bacterial communities, which remains largely unclear [8]. Nevertheless, it is known that phyllospheric microorganisms play a significant role in leaf functions, apical growth and flowering, seed mass, and the development of fruit [190]. It is not yet known how plants and microorganisms influence each other, how they exchange information, which metabolites are involved, and how the numerical and species composition of the microbiota is regulated depending on the plant species and age. Some aspects of selective leaf repopulation by specific genotype have been established [191]. However, it is not yet possible to summarize how this affects plant health and productivity and what the mechanisms regulating plant-bacterial relationships are, hindering answers to the fundamental question of how this regulation can be effectively used to benefit plant speed breeding technology.

## 8. Discussion and Problems

Speed breeding is a rapid-generation advancement technology that can accelerate crop improvement by optimizing growth conditions to achieve rapid generation turnover. The development of this technology holds great promise for solving various agricultural problems and global food and nutrition security challenges [23], representing a significant advancement in breeding acceleration. Plant–microbe interactions are not yet sufficiently understood to confidently leverage for stimulating plant growth and development under speed breeding conditions. Nevertheless, there is no doubt that the use of plant microflora should be included, especially when it comes to the efficiency and cost-effectiveness of speed breeding. Given multifactorial conditions and complex trait variation, AI-based systems for monitoring the soil-plant-atmosphere continuum [33,64]—mandatorily including microorganisms—represent a promising tool for identifying desired genotypes under speed breeding conditions (Figure 2).

**Figure 4:**
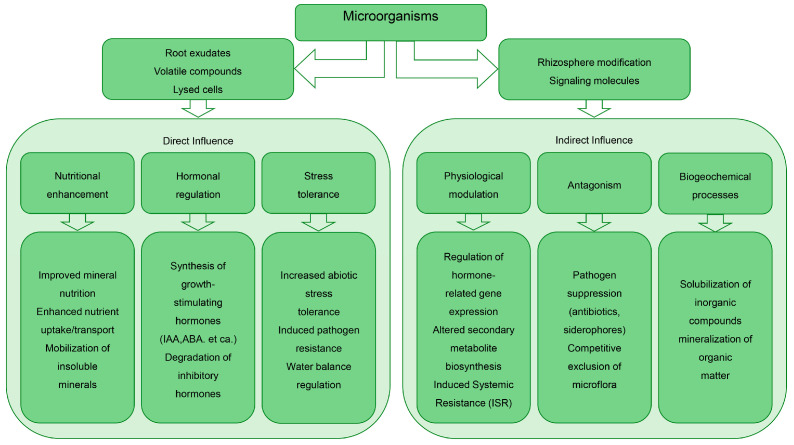
Microbial mechanisms enhancing plant growth in speed breeding. Microorganisms optimize plant development through direct and indirect interactions, including hormonal regulation, stress tolerance, nutrient enhancement, and pathogen suppression. These multifaceted roles collectively accelerate breeding outcomes in controlled environments.

Plants and their associated microorganisms form coevolved communities consisting of bacterial, archaeal, and a variety of eukaryotic species [192], where beneficial interactions between the plant host and its microbiome contribute to the overall health and optimal function of the holobiont (Figure 4).

Microorganisms can stimulate plant germination, growth, disease prevention, and overall fitness and stress tolerance. They fix atmospheric nitrogen and supplement plants with phosphorus, potassium and other nutrients from the soil, thereby improving plant growth and yields. Additionally, they increase trace element (Fe, Zn, and Se) availability via solubilization, chelation, and redox reactions [193], and secrete antioxidants, exopolysaccharides, and bioactive compounds (vitamins, hormones, and enzymes). These substances can directly affect plants by stimulating growth and increasing crop yields. In addition, they can help increase the uptake of water, nutrients, and essential elements, beneficially influencing plants [194,195,196,197]. Indirect mechanisms include pathogen inhibition and defense against various abiotic stresses through the production of biocontrol agents such as antibiotics and enzymes.

Emerging evidence suggests that stable productivity in speed breeding systems may be facilitated by dynamic interactions between plants, their associated microbiomes, and controlled environments, though microbiome-specific contributions under accelerated generational cycles require further mechanistic validation [113,116,118,147]. While microbial roles in nutrient provisioning [118], stress resilience [72], and growth promotion [156] are well-established in conventional agriculture, their functional optimization for speed breeding remains an active research frontier. However, in modern breeding work, insufficient attention has been given to improving the efficiency of interactions between plants and beneficial soil microorganisms. Speed breeding conditions are another factor that has favorable effects on growth and yield, as well as on the reproduction rate and speed of the entire plant development cycle. Critically, on the one hand, speed breeding in controlled environments represents a quick opportunity to obtain the maximum number of generations in a short period of time, and on the other hand, it involves modeling and programming the growth conditions to which the grown plants should be adapted as a result of this breeding, with the possibility of their further transfer to natural conditions. Within mutualistic systems, the plant-microorganism pair characterized by the plant’s longer life cycle and relative genetic stability forms the central governing element [198,199]. Thus, a well-selected beneficial soil microbiome acts as an essential component, supporting high yields under speed breeding’s stringent requirements. Using microbiological preparations based on plant-growth-promoting microorganisms [200,201,202] not only accelerates yield but also improves quality, reduces chemical inputs, and lowers agrochemical costs. Symbiotic nitrogen-fixing microorganisms and arbuscular mycorrhizal fungi are particularly important candidates for speed breeding within these mutualistic systems. Consequently, mutualistic symbioses involving valuable nodule bacteria, arbuscular mycorrhizal fungi, and various beneficial rhizosphere bacteria (epiphytes and endophytes) provide plants with enhanced capacity for accelerated growth and maturation under expedited cultivation in speed breeding [18,19,20,21,22].

The efficacy of biopreparations in practice is often limited, potentially due to competition and/or horizontal gene transfer between indigenous microflora and introduced strains [202,203]. Another significant reason may be the inability of many modern cultivated plant varieties to mutually benefit from beneficial soil microorganisms, since their selection was carried out against the background of high doses of mineral fertilizers and chemical plant protection agents [204]. Therefore, this point should be taken into account in both the breeding and further exploitation of plants.

Advances in molecular and cell biology have qualitatively improved the understanding of gene–metabolic integration in mutually beneficial plant-microbial systems. Assessing the practical need for beneficial soil microorganism complexes, especially for legume inoculation, is important. Breeding plants for enhanced microbe interaction potential within speed breeding systems is particularly crucial.

When microorganisms are used, the place that they are applied to on the plant is important, the roots or the top of the plant, as the effects may differ. This can be indicated, for example, by studies on the use of *Bacillus* to produce abscisic acid [17]. If they are applied to leaves, they stimulate water accumulation in the leaves, as they contribute to the closure of the stomata. At the same time, moving them to the roots has the opposite effect. In addition, it is likely necessary to consider seasonal dynamics and the demand for the active substances produced by microorganisms, which are necessary for plants at a certain stage of growth.

The use of endophytes and their metabolites can be considered as another possible promising research direction. Indeed, research in the field of endophytes is currently attracting increasing interest and may focus on crop management using transgenic modified endophytes in the near future [167]. One example of ongoing work in this area is the identification of novel endophytic strains with useful metabolic profiles [205], such as a previously unknown class of cyclic oligopeptides produced by endophytes [206,207]. In addition, previous studies have shown that associations formed with microorganisms [118] created by nature may be realistically used to influence the resulting yield of legume crops and their acquisition of new positive qualities.

Solutions based on the involvement of beneficial microorganisms are probably more versatile than the development of specific cultivars for growth in climatic chambers, as microorganisms have very high plasticity and can form associations with both homologous and nonhomologous hosts. In addition, microorganisms are versatile components of the cultivation medium and exhibit many other traits of interest to plants, in addition to increased tolerance under speed breeding conditions, making their use highly effective compared to chemical, genetic, or pure breeding approaches. However, the molecular mechanisms involved in such plant interactions are not yet sufficiently understood [95] to be able to exploit the full diversity of crops waiting to be used under speed breeding conditions. Plants have a direct impact on the environment and interact with microorganisms inhabiting the soil, plant surfaces (phyllosphere), and internal tissues (endosphere). This tripartite association (rhizosphere, phyllosphere, and endosphere) is vital for stable plant–microbe functioning and agricultural sustainability in any environment.

## 9. Conclusions and Future Perspectives

This review highlights the general principles and applications of using microorganisms in speed breeding technology to create the most favorable programmed conditions for stable results. From the review of the sources cited in this paper, it is evident that the microbiota plays an important role in plant growth, physiological health, and stress tolerance. High adaptability of the microbiota improves plant yield, physiological health, the production of regulatory compounds required for accelerated development and optimal plant health, biocontrol of phytopathogens, and stress tolerance under varying growing conditions. This provides the necessary margin of safety in transferring the resulting seeds of plants grown under rapid multiplication conditions to natural agroecosystems.

Accordingly, the most important conclusion of the work is that individually prepared microbial inoculant consortia can enhance speed breeding outcomes and plant traits, acting as an adaptation mechanism to subsequent growing conditions and increasing the importance of speed breeding technology for crop selection. To translate this potential into practice, future work should prioritize developing crop-specific microbial inoculants compatible with speed breeding protocols, ensuring seamless integration with controlled-environment growth parameters. This will require the individual selection of strains or consortia of microorganisms and their delivery at a specific period of plant development.

Because of the complexity of the question posed and the paucity of experimental data, whether sufficient and sustained acceleration of plant metabolism is possible under conditions of speed breeding, which relies on mutually beneficial neighboring microorganisms in interdependent conditions with the host, remains an open question. Future research may combine molecular techniques, genomic selection, epigenetics, and the latest technologies for detailed regulation of metabolic acceleration involving beneficial microorganisms to develop scalable, field-ready solutions, such as optimized microbial formulations for rapid-generation crops. The genetic editing of genes involved in symbiosis in plants and microorganisms, as a tool for speed breeding, will aim to improve their partnership efficiency.

Speed breeding technologies combined with optimized growth conditions to minimize generation time will continually improve breeders’ abilities to rapidly develop high-quality, resilient crop varieties, forming the backbone of the global food supply. Key translatable innovations include AI-driven microbial strain selection and automated delivery systems tailored to speed breeding environments, ensuring reproducibility across crops and conditions. Speed breeding bridges the gap between precision farming concepts and controlled breeding environments. Digital phenotyping can play a special role here as a tool for assessing microbiome–plant interactions under speed breeding conditions. The controlled environment of growth chambers allows time and costs to be precisely programmed. Realizing this enormous potential can significantly contribute to global food and nutritional security.

The use of microorganisms in speed breeding represents a powerful approach to accelerating growth, maintaining optimal plant conditions, and enabling rapid generation turnover. However, the controlled practical use of microorganisms for programmable outcomes under speed breeding remains nascent. Consequently, leveraging microorganisms to enhance speed breeding efficiency necessitates sustained and rigorous research to enable their efficient and precise application. Future efforts must focus on delivering translatable tools, such as modular microbial consortia kits for major crops, alongside standardized protocols for their deployment in speed breeding pipelines. These novel crop-specific strategies, including AI-based systems, will accelerate and improve the reliability of rapid generation turnover to achieve robust breeding outcomes.

## Data Availability

The data availability statement is not applicable.

## Abbreviations

The following abbreviations are used in this manuscript:

ABA	ABscisic Acid
ACC	1-AminoCyclopropane-1-Carboxylate
AI	Artificial Intelligence
AMF	Arbuscular Mycorrhizal Fungi
APX	Ascorbate Peroxidase
CAT	Catalase
EPSs	ExoPolySaccharides
GWAS	Genome-Wide Association Studies
IAA	Indole-3-acetic acid
MAS	Marker-Assisted Selection
NGS	Next-Generation Sequencing
PGPR	Plant-Growth-Promoting Rhizobacteria
SOD	Superoxide dismutase
QTLs	Quantitative Trait Loci
